# Comparison of the Effects of Norepinephrine and Phenylephrine Infusion in Preventing Hypotension during Spinal Anesthesia for Cesarean Delivery: A Randomized, Double-Blind Clinical Trial

**DOI:** 10.34172/aim.33931

**Published:** 2025-03-01

**Authors:** Saeed Jalili, Mitra Hojatansari, Somaye Abdollahi Sabet

**Affiliations:** ^1^Department of Anesthesiology, School of Medicine, Ayatollah Mousavi Hospital, Zanjan University of Medical Sciences, Zanjan, Iran; ^2^Department of Emergency and Critical Care Nursing, School of Nursing and Midwifery, Zanjan University Sciences, Zanjan, Iran; ^3^Department of Community Medicine, Faculty of Medicine, Social Determinants of Health Research Center, Zanjan University of Medical Sciences, Zanjan, Iran

**Keywords:** Cesarean delivery, Hypotension, Norepinephrine, Phenylephrine, Spinal anesthesia, Vasopressors

## Abstract

**Background:**

Hypotension following spinal anesthesia is one of the most common complications of cesarean delivery, posing significant risks to both maternal and fetal health. The use of vasopressors is a primary method for the prevention and management of hypotension.

**Objective:**

This study aimed to compare the efficacy of norepinephrine and phenylephrine infusion in preventing hypotension in patients undergoing cesarean section under spinal anesthesia.

**Methods:**

In this randomized, double-blind clinical trial, 90 pregnant women at 37 weeks of gestation scheduled for elective cesarean delivery were randomly assigned to receive either norepinephrine (n=47) or phenylephrine (n=43). The initial infusion rate was set at 5 µg/min for norepinephrine (up to a maximum of 60 mL/min) and 0.5 mg/min for phenylephrine (up to a maximum of 60 mL/min). Hemodynamic parameters, including systolic blood pressure (SBP) and diastolic blood pressure (DBP), mean arterial pressure (MAP), and heart rate, were assessed. Additionally, umbilical cord blood gas values (PACO_2_ and pH) at the time of birth were measured. Statistical analysis was performed using SPSS version 18 with descriptive statistics and independent t-tests or Mann-Whitney U tests (*P*≤0.05).

**Results:**

The findings revealed no statistically significant differences between the norepinephrine and phenylephrine groups regarding SBP and DBP, MAP, heart rate, and umbilical cord blood gas values (PACO_2_ and pH) at delivery (*P*≥0.05).

**Conclusion:**

Norepinephrine and phenylephrine appear to have similar efficacy in preventing hypotension during cesarean delivery. Clinicians may select either drug based on the patient’s clinical conditions and preferences.

## Introduction

 Neuraxial anesthesia is considered a reliable and effective method for pain relief during childbirth.^1^ Cesarean delivery typically requires a sensory block at the T5 level. The incidence of spinal anesthesia-induced hypotension in pregnant women undergoing cesarean section can reach up to 80%.^2,3^ Maternal hypotension is a common complication during cesarean delivery under spinal anesthesia, caused by reduced venous return, decreased cardiac output, or lowered systemic vascular resistance.

 Severe hypotension can lead to adverse maternal outcomes, including nausea, vomiting, dizziness, and cardiovascular collapse; it is a major contributor to maternal mortality during neuraxial anesthesia. Additionally, it compromises placental perfusion, increasing the risk of fetal acidosis, hypoxia, and postnatal neurological injuries.^4^ Pregnant women with pre-existing intravascular volume deficits are at heightened risk of cardiovascular compromise, as sympathetic blockade can significantly reduce venous return to the heart. Therefore, effective prevention or management of maternal hypotension is a critical aspect of obstetric anesthesia care.^5^

 Studies indicate that hypotension following spinal anesthesia during cesarean delivery is associated with a reduction in sympathetic tone within the arterial system. Consequently, the primary factor contributing to maternal hypotension in spinal anesthesia is a decrease in systemic vascular resistance, rather than a reduction in central venous pressure due to increased venous capacity. Therefore, the use of vasopressors is considered the most effective method for preventing and managing hypotension.^5-7^

 Currently, phenylephrine is the first-line treatment for hypotension following spinal anesthesia. Phenylephrine is a pure alpha-1 adrenergic receptor agonist that induces dose-dependent vascular constriction, with a predominant effect on veins rather than arteries. It also enhances venous return following sympathetic blockade.^8^ However, potential adverse effects include impaired peripheral blood flow in predisposed individuals, bradycardia, and fetal acidosis in pregnant women.^9^

 Although the efficacy of phenylephrine in managing hypotension following spinal anesthesia has been well-documented, recent studies highlight an ongoing debate about its selectivity as the preferred vasopressor.^10^ The primary cause of hypotension associated with spinal anesthesia is sympathetic blockade, and several strategies have been proposed for its management. These include slight head-down positioning, preloading with intravenous fluids before the block, and the use of sympathomimetic agents such as ephedrine and phenylephrine.^11,12^

 Phenylephrine’s effects are dose-dependent and can result in bradycardia, subsequently reducing cardiac output.^9^ This effect may occur even at low doses when blood pressure falls below baseline levels.^10^ Maternal cardiac output, compared to maternal blood pressure, has a closer correlation with uteroplacental blood flow.^11^

 In contrast to phenylephrine, norepinephrine appears to have a neutral effect on cardiac output and heart rate. This is due to its weak beta-adrenergic properties, which exert positive chronotropic effects, counterbalancing the negative chronotropic effects of its strong alpha-adrenergic action.^12^

 A primary concern with the use of α-agonists is the potential reduction in uteroplacental blood flow.^6^ However, studies suggest that norepinephrine does not impact fetal arterial blood pressure and does not compromise fetal-placental circulation. It offers better maternal and neonatal hemodynamic outcomes compared to ephedrine and phenylephrine.^4,6^

 Despite this, research in the field has yielded mixed results. Some studies indicate that both intermittent bolus and infusion administration of phenylephrine and norepinephrine maintain blood pressure, though reflex bradycardia and decreased cardiac output are more commonly associated with phenylephrine administration.^13,14^ Other studies have reported no significant difference in blood pressure changes following spinal anesthesia between bolus doses of phenylephrine and norepinephrine.^15,16^

 Theoretically, norepinephrine may be more advantageous in certain clinical scenarios. For instance, in cases of reduced uteroplacental circulation, such as preeclampsia or maternal cardiac conditions, norepinephrine could enhance systolic cardiac function and cardiac output without increasing heart rate. This makes it a potentially better option for preventing hypotension and bradycardia following neuraxial anesthesia.^14^

 Given that both drugs are utilized to manage hypotension resulting from spinal anesthesia during cesarean delivery and the limited studies on norepinephrine’s use in obstetric patients, this study aimed to compare the effects of phenylephrine and norepinephrine in managing maternal hypotension during spinal anesthesia for cesarean delivery. The goal was to identify the more effective drug with fewer side effects for both the mother and the neonate.

## Materials and Methods

 This study was a double-blind, parallel-group clinical trial conducted on 90 pregnant women referred to Ayatollah Mousavi Hospital in Zanjan for elective cesarean delivery. Participants were selected based on the following criteria: age between 18 and 35 years, ASA (American Society of Anesthesiologists) physical status classification of class 1 or 2, candidates for elective cesarean delivery, term pregnancy ( ≥ 37 weeks gestation), singleton pregnancy with a live fetus, no history of cardiovascular disease, no initial bradycardia upon admission, absence of preeclampsia diagnosis, no history of diabetes mellitus, cerebrovascular disease, or coagulation disorders, no use of narcotics, psychoactive substances, or alcohol, no history of multiple sclerosis or myasthenia gravis, and no known hypersensitivity to anesthetic drugs. Participants were excluded from the study in case of any of the following: failure of spinal anesthesia, requiring conversion to general anesthesia, withdrawal of consent to continue participation, cardiac or respiratory arrest during the study, and excessive hemorrhage beyond normal limits during the cesarean procedure.

 This rigorous selection process ensured a homogeneous sample for evaluating the effects of phenylephrine and norepinephrine in managing maternal hypotension during spinal anesthesia.

 The sample size was determined based on the study by Yazdanpanah et al, using a 95% confidence level and 80% statistical power.^17^ A total of 90 participants were estimated, with 45 individuals in each group.

 Participants were selected using convenience sampling and then allocated to one of the two groups (phenylephrine group or norepinephrine group) using stratified block randomization with six-block designations (A and B). Each block was assigned a number from 1 to 6, and a random number without replacement was selected from this range. Based on the chosen number, the corresponding block was extracted, and participants were allocated to the intervention group (norepinephrine) or the control group (phenylephrine) according to the block’s arrangement (the sequence of A and B in each block).

**Figure 1:**
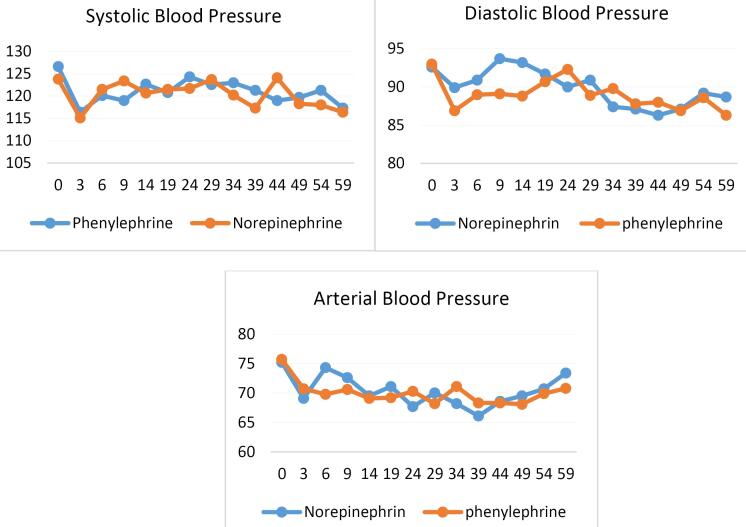
Changes in Mean Blood Pressure in the Phenylephrine and Norepinephrine Groups

 Each face of the block was labeled with a number, which was linked to the blocks in the randomization table to indicate the respective drug group assignment. This ensured balanced distribution across the groups while maintaining randomization integrity (Figure 1).

###  Blinding

 To ensure blinding, the researcher responsible for data collection identified patients by unique codes and was unaware of their group assignment. Similarly, the patients, despite signing an informed consent form acknowledging participation in the study, were blinded to their intervention group. The infusion drugs were prepared in coded syringes, ensuring that the anesthesiologist was also unaware of the drug administered.

###  Data Collection

 Data were collected using a researcher-designed questionnaire and checklist, reviewed and approved by 10 faculty members from the Anesthesiology Department. Blood pressure was measured in the operating room using a monitoring device. Umbilical cord blood samples from all participants were analyzed by a single laboratory technician using standardized protocols.

###  Study Procedure

 Following a detailed explanation of the study’s objectives and methods, the participants provided their informed consent. Term pregnant women ( ≥ 37 weeks) scheduled for elective cesarean section were randomly assigned to one of two groups to receive either norepinephrine or phenylephrine infusions.

 An 18G intravenous cannula was inserted into the antecubital vein. Patients were placed in a supine position, and baseline hemodynamic parameters, including non-invasive blood pressure, heart rate, and oxygen saturation, were measured three times and averaged.

 Participants underwent spinal anesthesia using a 25G spinal needle and 1.5-inch length, with 12.5 mg of 0.6% hyperbaric bupivacaine injected into the L4-L5 or L3-L4 space using the median approach after proper antiseptic preparation. Following spinal anesthesia, 10 mL/kg of lactated Ringer’s solution was infused, with a maximum infusion of 3 liters during the procedure.

 Once the patient was supine, the intervention drug infusion commenced. Norepinephrine was initiated at 5 µg/min (maximum 60 mL/min), and phenylephrine at 0.5 mg/min (maximum 60 mL/min). Vital signs, including heart rate, systolic blood pressure (SBP), diastolic blood pressure (DBP), and mean arterial pressure (MAP), were monitored every minute from infusion onset until neonatal delivery and then every five minutes until the surgery concluded.

 Umbilical cord blood gas analysis, including pH, base excess, and PCO_2_, was conducted using a specialized kit (GEM Premier 3000, Instrumentation Laboratory, Bedford, Massachusetts).

###  Statistical Analysis

 Descriptive statistics and independent *t* tests were used to compare mean hemodynamic parameters and umbilical cord blood gases between the norepinephrine and phenylephrine groups. For non-normally distributed data, the Mann-Whitney U test was applied. All analyses were performed using SPSS version 18, with the significance level set at 0.05.

## Results

 The findings of this study revealed no statistically significant differences in the demographic characteristics of participants between the two groups. The groups were comparable in terms of age, weight, height, and body mass index (BMI) (*P* value ≥ 0.05) (Table 1).

**Table 1:** Comparison of Mean Values (SD) of Vital Sign in the Two Intervention Groups

**Time After Anastasia (min)**	**Intervention Group **	**Systolic Blood Pressure**		**Diastolic Blood Pressure**		**Arterial Blood Pressure **		**Pulse Rate**	
		**Mean (SD)**	***P *** Value^*^	**Mean (SD)**	***P *** Value^*^	**Mean (SD)**	***P *** Value^*^	**Mean (SD)**	***P *** Value^*^
Base time	Norepinephrine	128.5 (11.9)	0.65	83.5 (7.9)	0.81	95.7 (12.1)	0.51	95.7 (15.5)	0..82
	Phenylephrine	124.9 (10.9)		81.3 (8.3)		95.3 (11.5)		93.0 (11.7)	
0	Norepinephrine	123.8 (15.4)	0.89	75.2 (14.5)	0.61	92.6 (15.5)	0.63	87.6 (12.9)	0.89
	Phenylephrine	126.6 (15.9)		75.7 (13.0)		93.0 (11.7)		86.0 (14.2)	
3	Norepinephrine	115.1 (22.2)	0.62	69.1 (15.9)	0.55	89.9 (17.5)	0.51	73.2 (13.2)	0.62
	Phenylephrine	116.4 (20.0)		70.7 (14.3)		86.9 (16.6)		83.7 (11.6)	
6	Norepinephrine	121.5 (24.1)	0.74	74.3 (15.6)	0.57	90.9 (14.9)	0.61	88.3 (12.8)	0.74
	Phenylephrine	120.1 (23.2)		69.8 (16.6)		89.0 (17.4)		90.0 (13.3)	
9	Norepinephrine	123.4 (24.5)	0.82	72.6 (18.0)	0.60	93.7 (20.2)	0.55	80.7 (12.8)	0.82
	Phenylephrine	119.8 (16.4)		70.6 (14.1)		89.1 (14.3)		85.4 (11.3)	
14	Norepinephrine	120.7 (17.8)	0.63	69.5 (13.8)	0.51	93.2 (15.1)	0.87	95.4 (13.1)	0.63
	Phenylephrine	122.7 (15.7)		69.1 (13.6)		88.8 (14.1)		93.8 (12.5)	
19	Norepinephrine	121.5 (14.6)	0.58	71.1 (12.1)	0.57	91.7 (15.3)	0.77	87.7 (11.3)	0.58
	Phenylephrine	120.8 (15.1)		69.2 (11.8)		90.7 (10.9)		88.7 (12.9)	
24	Norepinephrine	121.7 (14.4	0.91	67.7 (13.9)	0.66	90.0 (13.7)	0.52	76.0 (14.5)	0.91
	Phenylephrine	124.3 (15.5)		70.3 (12.8)		92.3 (12.8)		79.3 (11.8)	
29	Norepinephrine	123.7 (14.8)	0.66	70.0 (12.3)	0.61	90.9 (13.8)	0.63	94.5 (12.0)	0.66
	Phenylephrine	122.6 (12.3)		68.2 (12.4)		88.9 (12.6)		96.2 (11.6)	
34	Norepinephrine	120.2 (14.2)	0.74	68.2 (13.7)	0.53	87.4 (12.7)	0.57	83.4 (14.7)	0.74
	Phenylephrine	123.0 (13.1)		71.1 (11.4)		89.8 (12.2)		88.1 (15.2)	
39	Norepinephrine	117.3 (14.7)	0.66	66.1 (14.0)	0.57	87.1 (14.3)	0.89	82.1 (13.8)	0.66
	Phenylephrine	121.3 (13.0)		68.3 (13.6)		87.8 (12.1)		82.8 (14.1)	
44	Norepinephrine	124.1 (16.7)	0.58	68.6 (13.9)	0.55	86.3 (14.3)	0.69	90.8 (12.3)	0.58
	Phenylephrine	119.0 (14.5)		68.3 (11.0)		88.0 (11.0)		92.9 (11.6)	
49	Norepinephrine	118.7 (14.8)	0.58	69.5 (13.3)	0.61	87.1 (12.9)	0.59	85.0 (16.6)	0.59
	Phenylephrine	119.7 (14.1)		68.1 (11.1)		86.9 (12.1)		81.9 (13.4)	
54	Norepinephrine	118.3 (17.2)	0.63	70.7 (13.4)	0.52	89.2 (14.4)	0.63	83.6 (15.3)	0.63
	Phenylephrine	121.3 (11.3)		69.9 (10.6)		88.6 (11.0)		82.3 (12.6)	
59	Norepinephrine	116.4 (14.7)	0.66	73.4 (12.0)	0.63	88.7 (13.4)	0.66	84.2 (13.9)	0.66
	Phenylephrine	117.8 (13.7)		70.8 (11.0)		86.3 (11.6)		86.3 (11.6)	

*Mann-Whitney & independent *t* test.

 Regarding hemodynamic variables, the mean values of SBP (*P* = 0.65), DBP (*P* = 0.72), MAP (*P* = 0.72), and heart rate (*P*= 0.67) at the onset of anesthesia and other time points showed no statistically significant differences between the phenylephrine and norepinephrine groups (*P* value ≥ 0.05) (Table 2 and Figure 1).

**Table 2:** Comparison of Mean Values (SD) of the Assessed Parameters in the Two Intervention Groups

**Variables**	**Intervention Groups**	**Mean (SD)**	***P *** Value*
Systolic blood Pressure	Norepinephrine	13.2)) 123.1	0.65
	Phenylephrine	119.5 (13.6)	
Diastolic blood Pressure	Norepinephrine	75.2 (11.4)	0.72
	Phenylephrine	74.9(10.7)	
Arterial blood Pressure	Norepinephrine	88.6(8.9)	0.72
	Phenylephrine	90.2(10.7)	
Pulse rate	Norepinephrine	83.9(18.3)	0.67
	Phenylephrine	86.0(10.5)	
PACO_2_ Umbilical blood	Norepinephrine	(11.4) 48.5	0.79
	Phenylephrine	44.7(10.5)	
Umbilical blood pH	Norepinephrine	0.54(2.1)	0.52
	Phenylephrine	0.69(1.9)	

*Mann-Whitney test.

 Additionally, no significant differences were observed between the two groups concerning mean umbilical cord blood gas parameters, including PACO_2_ (*P* = 0.79) and umbilical cord blood pH (*P* = 0.52), immediately after delivery (Table 2).

## Discussion

 In this study, no statistically significant differences were observed between the phenylephrine and norepinephrine groups in terms of systolic and DBP, MAP, and heart rate. This suggests that neither drug is superior to the other in preventing hypotension during cesarean delivery.

 The findings of this study align with the results of Mohta et al.^15^ However, in the study by Yazdanpanah et al, norepinephrine was associated with a lower percentage of blood pressure reduction and a smaller decrease in heart rate compared to phenylephrine.^17^

 Similarly, studies by Ngan Kee et al and Wang et al reported higher cardiac output five minutes after spinal anesthesia in the norepinephrine group compared to the phenylephrine group, along with fewer occurrences of bradycardia and reduced cardiac output.^4,6^

 Additionally, Vakili et al and Mon et al demonstrated that while phenylephrine effectively maintained SBP, it was associated with reduced maternal heart rate and cardiac output.^18,19^

 In studies conducted by Cho et al, Hasanin et al, and Sharkey et al, which compared the effects of intermittent bolus and infusion of phenylephrine and norepinephrine on blood pressure and heart rate, both drugs-maintained blood pressure effectively. However, the phenylephrine group exhibited more reflex bradycardia and reduced cardiac output.^13,14,16^

 The discrepancies in these study results may be attributed to variations in the dosage of drugs used, methods of drug administration, monitoring tools, and techniques for tracking hemodynamic changes.

 Alpha-agonists are considered the first-line vasopressors for use during caesarean sections. However, phenylephrine has been associated with a reflex decrease in heart rate, which may lead to a reduction in cardiac output. Studies have shown that heart rate changes are closely linked to cardiac output, with heart rate serving as the best surrogate indicator of cardiac output during caesarean delivery.^20^ There is also a suggested correlation between cardiac output and uteroplacental blood flow.^21^ Noradrenaline, due to its weak β-agonist activity, is thought to cause a smaller decrease in heart rate and may better preserve cardiac output.^22^ Additionally, noradrenaline is hypothesized to have a reduced cardiac inhibitory effect because of its mild beta-adrenergic agonistic activity alongside alpha-adrenergic activity.^23^

 In this study, no statistically significant difference was observed in the mean PACO_2_ and umbilical cord blood pH between the norepinephrine and phenylephrine groups. Other studies align with the present study in this regard.^13-15^ In the study by Mohta et al, PACO_2_ levels in umbilical cord blood did not show a significant difference between the two groups; however, the pH of umbilical cord blood was significantly higher in the phenylephrine group (pH = 7.29) compared to the norepinephrine group (pH = 7.25).^15^ This discrepancy may be attributed to the higher dose of phenylephrine used in that study. Additionally, in the study by Ngan Kee et al, umbilical cord blood pH was higher in the norepinephrine group,^4^ possibly due to the transfer of blood flow to the placenta and stimulation of fetal metabolism mediated by β-adrenergic agonists.^24^

 The limitations of this study include limitations in obtaining arterial and central venous catheters, lack of central catheterization, and devices for measuring cardiac output through arterial catheters, along with patients’ unwillingness to undergo spinal anesthesia. A strength of this study is the achievement of favorable results despite the use of lower doses of vasopressors and bolus infusion to prevent peripheral ischemia.

## Conclusion

 In conclusion, 100 μg phenylephrine and 5 μg noradrenaline boluses had similar efficacy for treatment of hypotension in patients undergoing elective caesarean section under spinal anesthesia, with no difference in the incidence of maternal blood pressure and bradycardia and umbilical blood PACO_2_ and pH.

 According to this study, conducting new research using innovative, non-invasive methods for measuring cardiac output and stroke volume is recommended. These methods could provide more precise information regarding the effects of norepinephrine, leading to a deeper understanding of its impact on hemodynamic parameters and maternal and neonatal outcomes.
